# Pulmonary vein stenosis after pediatric heart transplantation: incidence and risk factors

**DOI:** 10.3389/fsurg.2026.1820935

**Published:** 2026-05-28

**Authors:** Hyun Soo Lee, Han Ki Park, Sang On Lee, Yu Rim Shin

**Affiliations:** Division of Cardiovascular Surgery, Department of Thoracic and Cardiovascular Surgery, Severance Cardiovascular Hospital, Yonsei University College of Medicine, Seoul, Republic of Korea

**Keywords:** congenital heart disease, heart transplant, pediatric heart transplantation, predicted heart mass, pulmonary vein stenosis

## Abstract

**Objectives:**

Postoperative pulmonary vein stenosis (PVS) is an uncommon but potentially serious complication following pediatric heart transplantation. This study aimed to determine the prevalence of PVS and identify donor- and recipient-related predictors.

**Methods:**

We retrospectively reviewed pediatric patients (<18 years) who underwent heart transplantation at a single center between 2003 and 2024. PVS was defined as a ≥ 4 mmHg Doppler gradient across any pulmonary vein on echocardiography or significant narrowing on computed tomography. Donor and recipient variables-including congenital heart disease (CHD), predicted heart mass (PHM), donor–recipient size/sex were analyzed using univariate and multivariable logistic regression. The primary endpoint was the occurrence of PVS; the secondary endpoint was overall mortality.

**Results:**

Among 50 recipients (median age, 11 years; range, 0.2–17 years), 9 (18%) developed *de novo* PVS with a median time to diagnosis of 84 days (range, 4–1088 days). On univariate analysis, CHD (*p* = 0.007), PHM (*p* = 0.017), and male-to-female donor–recipient mismatch (*p* = 0.035) were significantly associated with PVS. In multivariate analysis, CHD remained the only associated factor (*p* = 0.015; OR, 11.25; 95% CI, 1.60–78.98). PVS did not significantly affect survival probability (*p* = 0.240).

**Conclusions:**

PVS occurred in nearly one-fifth of pediatric heart transplant recipients, predominantly in those with CHD. However, given the limited sample size, these findings should be considered exploratory and hypothesis-generating. Donor–recipient size mismatch parameters were consistently greater among patients with PVS, though this association warrants validation in larger prospective cohorts. Although PVS was not associated with increased mortality, identification of high-risk groups remains essential for surveillance and preventive strategies in pediatric heart transplantation.

## Introduction

Pulmonary vein stenosis (PVS) after pediatric heart transplantation (HTx) is an uncommon but clinically significant complication. Although HTx aims to normalize hemodynamics, PVS can develop *de novo* or progress postoperatively, leading to pulmonary hypertension, graft dysfunction, and adverse outcomes (1, 2). PVS in general is characterized by progressive neointimal proliferation and obstruction of pulmonary venous return. The prognosis of progressive PVS remains poor, with reported two-year survival rates as low as 50%–60% in infants (3–6). Multivessel involvement and earlier age at diagnosis are particularly associated with worse outcomes. Several risk factors for PVS have been described, including congenital heart disease (CHD), prematurity, and bronchopulmonary dysplasia, as well as surgical manipulation at the pulmonary venous–atrial junction (7, 8). However, mechanisms remain incompletely understood, and the disease course is unpredictable. Recent multicenter and single-center studies report that 4%–20% of pediatric HTx recipients develop PVS (1–4). CHD is consistently identified as the strongest risk factor, while donor–recipient size mismatch and technical considerations may also contribute (3, 4). Although recent studies have identified CHD as a potential risk factor for PVS, long-term data from single-center cohorts remain limited, and the specific impact of donor–recipient size mismatch is yet to be fully validated. Therefore, evaluating the precise incidence and refined predictors in a longitudinal pediatric HTx cohort is essential to optimize graft survival and establish evidence-based surveillance strategies.

## Patients and methods

We retrospectively reviewed all pediatric patients (<18 years) who underwent orthotopic heart transplantation at our institution between January 2003 and March 2024. We defined the endpoint as newly developed PVS after transplantation; pre-existing PVS was not counted as an event. Clinical data were obtained from electronic medical records. PVS was defined as (1) a Doppler echocardiographic gradient ≥ 4 mmHg across any pulmonary vein or (2) significant luminal narrowing on contrast-enhanced cardiac computed tomography. Echocardiographic evaluations were reviewed by a pediatric cardiologist, and cardiac CT scans were reviewed by a radiologist. *De novo* PVS was defined as a newly developed obstruction of one or more pulmonary veins after HTx in patients with no evidence of pre-existing pulmonary venous pathology. To confirm the absence of pre-transplant PVS, all patients underwent a comprehensive multi-modal evaluation prior to transplantation, including high-resolution CT and transthoracic echocardiography. Clinically significant PVS was defined as cases requiring intervention or resulting in PVS-attributed mortality. In cases of complex CHD, diagnostic cardiac catheterization was performed to assess pulmonary vascular resistance and anatomy prior to transplantation. Post-HTx surveillance was performed according to a standardized, multi-modal imaging protocol. Transthoracic echocardiography including Doppler interrogation of all four pulmonary vein orifices, and CT are routinely performed at 1 month, 6 months, and 12 months post-transplantation, and annually thereafter.

Patients were grouped according to the occurrence of PVS. Recipient characteristics included age, sex, birth history, underlying cardiac diagnosis, and preoperative status were compared between groups. Donor–recipient mismatch variables included predicted heart mass (PHM) ratio, donor-to-recipient weight and body surface area (BSA) ratio, and sex mismatch (male-to-female, female-to-male). The primary outcome was the occurrence of PVS. The secondary outcome was all-cause mortality. Continuous variables are expressed as medians with interquartile ranges and compared using the Mann–Whitney U test. Categorical variables were analyzed using the chi-square or Fisher's exact test. Variables with *p* < 0.05 in univariate analysis were entered into multivariate logistic regression. Odds ratios (ORs) and 95% confidence intervals (CIs) were reported. A *p*-value < 0.05 was considered statistically significant. The Mantel-Cox log-rank test was employed to analyze survival outcomes for the PVS and non-PVS groups. Analyses were performed using SPSS (IBM Corp., Armonk, NY). This study was reviewed and approved by the Institutional Review Board of Severance Hospital, Yonsei University Health System (IRB No. 2025-2656-002). The requirement for informed consent was waived due to the retrospective nature of the study.

## Results

### Incidence and clinical characteristics

A total of 50 pediatric patients underwent heart transplantation during the study period. Median age was 11 years (range, 0.2–17 years). 60% of the patients were male. Median follow up time was 4.2 years (range, 0–22.1 years). The baseline characteristics of the study population, categorized by the occurrence of PVS, are summarized in Table 1. Nine patients (18%) developed *de novo* PVS. The median time to PVS occurrence was 84 days (range, 4–1088 days). Among the nine patients (18%) who developed *de novo* PVS, the clinical course varied significantly. We categorized these cases into clinically significant PVS (*n* = 2, 4%) and subclinical PVS (*n* = 7, 14%). (Supplementary Table S1) Clinically significant PVS, defined by the need for intervention or PVS-attributed mortality, occurred exclusively in the two patients with bilateral involvement. One of these patients exhibited a highly progressive clinical course, requiring surgical repair followed by five separate transcatheter interventions, and ultimately died from PVS-related complications 3 months post-transplant. The second patient required a single session of stent insertion and angioplasty 2 months post-transplant and has since remained stable. Both patients demonstrated significant donor-recipient size mismatch (weight ratios of 1.94 and 2.81, respectively). In contrast, the remaining seven patients (14%) were classified as having subclinical PVS. These cases involved isolated unilateral stenosis (right PV in 6, left PV in 1) and were characterized by a stable clinical course. None of these patients exhibited disease progression or required surgical or transcatheter intervention during the follow-up period.

**Table 1:** Baseline patient characteristics .

Variable	No PVS (*N* = 41)	PVS (*N* = 9)	*p*-value^a^
Age, years	12 (9–16)	7 (2–11)	0.156
Male sex, n(%)	29 (70.7)	3 (33.3)	0.034
Congenital heart disease, n(%)	10 (24.3)	7 (77.7)	0.002
DORV	1	1	
Unbalanced AVSD	0	1	
Pulmonary atresia with VSD	0	2	
TGA	2	1	
Congenital aortic stenosis	2	0	
TAPVR	1	0	
TOF	1	0	
TA/TS/Ebstein anomaly	2	1	
Congenital MR	1	0	
CoA with VSD	0	1	
Cardiomyopathy, n(%)	30 (73.2)	3 (33.3)	0.477
Dilated, n(%)	19	3	
Restrictive	3	0	
ARVD	1	0	
Myocarditis	7	0	
Low birth weight or prematurity, n(%)	4 (9.7)	1 (11.1)	0.902
Donor weight, kg	60.0 (55.0–76.0)	31.0 (19.6–73.3)	0.012
Recipient weight, kg	38.0 (24.6–53.0)	16.6 (13.8–38.0)	0.021

PVS, pulmonary vein stenosis; DORV, double outlet right ventricle; AVSD, atrioventricular septal defect; VSD, ventricular septal defect; TGA, transposition of the great arteries; TAPVR, total anomalous pulmonary venous return; TOF, tetralogy of fallot; TA, tricuspid atresia; TS, tricuspid stenosis; MR, mitral regurgitation; CoA, coarctation of the aorta.

a Variables are reported as median (interquartile range) or number (%). Continuous variables were compared using the Mann–Whitney U test and categorical variables using the Chi-square or Fisher's exact test as appropriate.

b Categories are not mutually exclusive.

**Figure 1:**
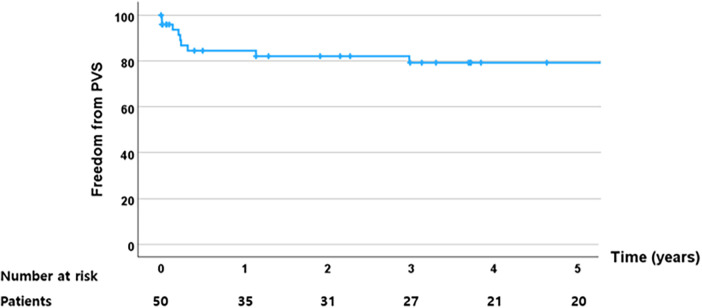
Kaplan–meier curve of freedom from pulmonary vein stenosis.

Kaplan–Meier analysis indicated that freedom from PVS was approximately 85% at 1 year, 79% at 3 years, and remained stable thereafter, suggesting that most events occur within the early postoperative phase (Figure 1).

### Mortality: impact of pulmonary vein stenosis

Regarding overall outcomes, 12 deaths occurred during follow-up. (Table 2) Among the three deaths in the PVS group, one was directly attributed to PVS-related complications; this patient developed secondary pulmonary hypertension and irreversible pulmonary injury, ultimately succumbing to sepsis while awaiting heart–lung transplantation. This patient was diagnosed with PVS 3 months after transplantation. At the time of the heart transplant, the recipient was 2 months old. Regarding the donor-size mismatch, the donor-to-recipient weight ratio was 1.94, and the PHM ratio was 1.76, indicating a significant size mismatch. In contrast, the remaining two deaths in the PVS group were unrelated to PVS, involving chronic rejection (*n* = 1) and sepsis (*n* = 1). There was no significant difference in survival between the PVS and non-PVS groups (Log-rank test, *p* = 0.240).

**Table 2:** Causes of mortality during follow-up .

Cause of Death	No PVS (*n* = 9)	PVS (*n* = 3)	Total (*n* = 12)
PVS-related death	0 (0%)	1 (33.3%)	1 (8.3%)
Rejection	5 (55.6%)	1 (33.3%)	6 (50.0%)
Acute rejection	4	0	4
Chronic rejection	1	1	2
Sepsis	3 (33.3%)	1 (33.3%)	4 (33.3%)
Intracranial hemorrhage	1 (11.1%)	0 (0%)	1 (8.3%)

PVS, pulmonary vein stenosis.

### Risk factors for pulmonary vein stenosis

Logistic regression analysis was performed to identify potential predictors of *de novo* PVS. In univariate analysis, CHD was strongly associated with the development of PVS (OR 10.85; 95% CI 1.93–60.93; *p* = 0.007). Among donor-recipient mismatch variables, a higher PHM ratio (OR 36.27; 95% CI 1.92–687.05; *p* = 0.017) and male donor to female recipient sex mismatch (OR 5.16; 95% CI 1.12–23.69; *p* = 0.035) were also significant predictors. Other size-related parameters, including the donor-to-recipient weight ratio (*p* = 0.094) and BSA ratio (*p* = 0.080), showed potential associations but did not reach statistical significance in the univariate model. Variables that demonstrated significance in the univariate analysis were included in a multivariate logistic regression model to explore potential associated factors. In this model, CHD remained the only associated factor for post-transplant PVS (OR 11.25; 95% CI 1.60–78.98; *p* = 0.015). Although the PHM ratio showed a strong trend toward an association with PVS, it did not reach statistical significance in the multivariate analysis (OR 40.78; 95% CI 0.79–2103.81; *p* = 0.065) (Table 3).

**Table 3:** Risk factors for pulmonary vein stenosis.

Variable	Univariate analysis	Multivariate analysis
Age, years	OR 0.90 (95% CI: 0.80–1.02), *p* = 0.107	
Congenital heart disease	OR 10.85 (95% CI: 1.93–60.93), *p* = 0.007	OR 11.25 (95% CI: 1.60–78.98), *p* = 0.015
Male donor to female recipient	OR 5.16 (95% CI: 1.12–23.69), *p* = 0.035	OR 1.78 (95% CI: 0.28–11.27), *p* = 0.540
Female donor to male recipient	OR 0.89 (95% CI: 0.16–4.97), *p* = 0.890	
Predicted heart mass ratio	OR 36.27 (95% CI: 1.92–687.05), *p* = 0.017	OR 40.78 (95% CI: 0.79–2103.81), *p* = 0.065
Donor to recipient weight ratio	OR 2.91 (95% CI: 0.83–10.16), *p* = 0.094	
Donor to recipient BSA ratio	OR 6.19 (95% CI: 0.80–47.82), *p* = 0.080	

OR, odds ratio.

### Donor-Recipient mismatch profiles

The donor-to-recipient mismatch variables are detailed in Table 4. The PVS group exhibited significantly higher discrepancies in size-related parameters. Specifically, the PHM ratio was significantly elevated in the PVS group (1.33 vs. 1.09, *p* = 0.013), as was the donor-to-recipient body BSA ratio (1.54 vs. 1.26, *p* = 0.030). Furthermore, the male donor to female recipient combination was more prevalent among patients who developed PVS (55.5% vs. 19.5%, *p* = 0.026).

**Table 4:** Donor-Recipient mismatch variables.

Variable	No PVS (*N* = 41)	PVS (*N* = 9)	*p*-value^a^
Male donor to female recipient, n(%)	8 (19.5)	5 (55.5)	0.026
Female donor to male recipient, n(%)	10 (24.3)	2 (22.2)	0.890
Donor to recipient predicted heart mass ratio	1.09 (0.94–1.32)	1.33 (1.23–1.61)	0.013
Donor to recipient weight ratio	1.52 (1.16–2.01)	1.94 (1.68–2.54)	0.063
Donor to recipient BSA ratio	1.26 (1.07–1.54)	1.54 (1.40–1.74)	0.030

BSA, body surface area.

a Variables are reported as median (interquartile range) or number (%). Continuous variables were compared using the Mann–Whitney U test and categorical variables using the Chi-square or Fisher’s exact test as appropriate.

## Discussion

In this study, we investigated the incidence and risk factors of PVS in pediatric heart transplant recipients over a 20-year period. The observed incidence of *de novo* PVS in our cohort was 18%, with the majority of cases diagnosed within the first year post-transplantation. This is relatively higher than the 4.5% to 10.5% reported in previous multicenter and single-center studies (2, 3); however, direct numerical comparison should be interpreted with caution, as these studies employed different surveillance strategies and imaging protocols. Notably, when restricting to clinically significant PVS—defined as cases requiring intervention or resulting in PVS-attributed mortality—the rate in our cohort was 4%, which is more comparable to the lower end of previously reported ranges. The higher overall detection rate likely reflects a significant detection bias inherent to our intensive, multi-modal surveillance strategy, which specifically mandates Doppler interrogation of all pulmonary vein orifices during every echocardiographic exam, supplemented by routine CT imaging. This proactive approach enabled the detection of even mild or subclinical stenosis that might have been underreported in studies focusing only on clinically overt cases. Additionally, the high proportion of infants and complex CHD cases in our center, combined with significant donor-recipient size mismatch, may have further contributed to the elevated incidence. While some identified cases were initially mild, their documentation is clinically important as they may serve as precursors to more extensive obstruction.Butto et al. also reported PVS after pediatric heart transplantation, noting that most cases were diagnosed at a median of 2 months post-transplant (2). Our median interval to diagnosis of 84 days strongly supports the emerging consensus that PVS is primarily an early postoperative phenomenon. Takajo et al. reported an incidence of approximately 10.5% using their institutional protocol (3). However, as surveillance strategies differed across studies, direct comparison of these rates should be made with caution. Therefore, our reported incidence should be interpreted as method-dependent and may represent the broader biological spectrum of pulmonary venous remodeling rather than only clinically significant disease.

CHD has been repeatedly highlighted as a predominant risk factor. In our multivariate analysis, CHD carried an 11-fold higher risk of PVS, a finding that is consistent with the results of Butto et al., where 16 out of 19 PVS patients had underlying CHD (2). This association may stem from the complex venous anatomy and the necessity for extensive surgical reconstruction at the pulmonary venous–atrial junction. As suggested by Choi et al., the surgical manipulation required to address distorted venous return in CHD patients may trigger a cascade of neointimal proliferation, leading to progressive luminal narrowing (1). A unique and noteworthy aspect of our study is the exploration of donor-recipient size mismatch as a potential contributor to PVS. Beyond the predicted heart mass (PHM) ratio, our analysis revealed that oversized donor parameters across multiple aspects—including weight and BSA ratios—consistently demonstrated a trend associated with the development of PVS. Although these parameters did not reach statistical significance in the multivariate model (*p* = 0.065 for PHM), the consistent trend observed in the univariate analysis is noteworthy. While size mismatch has been extensively studied in the context of primary graft function, its role in PVS is less defined in existing literature. Our findings suggest that donor-recipient size mismatch is a clinically interesting parameter that may merit further investigation. From a mechanistic perspective, a disproportionately large donor heart might exert extrinsic compression on the pulmonary veins or lead to unfavorable geometry, such as kinking at the anastomotic site, particularly within the constrained mediastinum of a small child. This hypothesis is supported by the observations of Butto et al., who noted that *de novo* PVS often involves the right pulmonary veins, which may be more susceptible to compression by an oversized graft (2). While the association between donor-recipient size mismatch and PVS is clinically intriguing, it remains speculative. This factor did not persist as a significant predictor in our multivariable model. Thus, we propose size mismatch as a potential contributing hypothesis rather than a confirmed causal mechanism, requiring further validation in larger, prospective cohorts. In terms of the impact of age at transplantation, our analysis did not show a statistically significant correlation with the development of PVS. However, it is noteworthy that all patients who developed PVS in our cohort were infants or young children under 9 months of age at the time of transplant. The lack of statistical significance may be attributed to the limited sample size of our study and the overall young age distribution of our pediatric HTx recipients, which might have masked the differential risk. Clinically, younger children, especially infants, are known to be at a higher risk for PVS due to smaller pulmonary vein diameters and the technical complexity of the anastomoses in a restricted mediastinal space. Furthermore, as shown in our cases, significant donor-size mismatch (oversizing) in these small infants may further compromise the pulmonary venous return by causing anatomical distortion or compression. Regarding clinical outcomes, our study found no significant association between PVS and overall mortality. Notably, PVS-attributed mortality occurred exclusively in the clinically significant subgroup (*n* = 2, 4%), while none of the subclinical cases contributed to mortality, which may partly explain the absence of a statistically significant survival difference between groups. This contrasts with some literature, such as Takajo et al., which suggested higher mortality in PVS patients during the early follow-up phase (3). The discrepancy may be due to our relatively small sample size or a high proportion of unilateral PVS cases that did not progress to global pulmonary hypertension. However, the case of one patient in our cohort who required five separate sessions of interventions underscores the significant morbidity and the “refractory” nature of PVS described by Choi et al. (1). The delayed manifestation of PVS, occurring months after OHT rather than immediately postoperatively, may be explained by the nature of the mechanical interaction between the donor heart and the recipient's mediastinum. While spatial constraints due to donor-size mismatch exist from the time of surgery, the resulting compression or anatomical distortion may initially be subtle. Over time, however, this persistent mechanical stress and altered hemodynamics likely trigger a progressive fibroproliferative response inside the pulmonary veins. This is consistent with previous studies characterizing PVS as a progressive lesion, where chronic intimal hyperplasia and remodeling lead to a gradual reduction in luminal diameter. Therefore, the observed delay in diagnosis reflects the time required for these secondary biological changes to manifest as clinically or radiologically significant stenosis.

## Limitations

This study has several limitations that should be considered when interpreting the results. First, its retrospective, single-center design may limit the generalizability of the findings to other institutions with different surgical techniques or post-transplant management protocols. Second, the relatively small sample size and the small number of PVS events may have reduced the statistical power, potentially masking the significance of certain risk factors in the multivariate analysis. Specifically, while the donor-recipient size mismatch parameters (PHM, weight, and BSA ratios) showed a strong consistent trend, they did not reach the threshold for statistical significance. Third, the lack of standardized timing for postoperative CT scans might have led to an underestimation of the true incidence or a delay in the diagnosis of asymptomatic PVS. As a single-center study with a specific surveillance protocol and a high proportion of complex CHD cases, the generalizability of our findings may be limited. Our results reflect the experience of a specialized tertiary center and may not be applicable to institutions with different patient demographics or surveillance strategies. Further prospective, multi-center studies with larger cohorts are warranted to better define the complex interplay between anatomical and clinical predictors of PVS.

## Conclusions

In conclusion, pulmonary vein stenosis is a significant and clinically challenging complication following pediatric heart transplantation, particularly in the early postoperative period. Our study identifies congenital heart disease as the strongest associated factor for *de novo* PVS, while also highlighting the potential role of donor-recipient size mismatch—including PHM, weight, and BSA—as a noteworthy clinical parameter. Although PVS was not directly associated with increased mortality in this cohort, the high morbidity and the need for repeated interventions in bilateral cases underscore the importance of early detection. Vigilant surveillance using echocardiography and a low threshold for cardiac CT are essential for high-risk recipients, especially those with CHD or oversized grafts, to ensure timely intervention and optimal graft preservation. Given the exploratory nature of these findings and the inherent limitations of our sample size, prospective multicenter validation is essential before these associations can inform clinical practice.

## Data Availability

The datasets generated during and/or analyzed during the current study are not publicly available due to patient confidentiality and institutional restrictions but are available from the corresponding author on reasonable request. Requests to access the datasets should be directed to dlgustn0204@naver.com.
