# Supplementary material for: Pulmonary vein stenosis after pediatric heart transplantation: incidence and risk factors
Source: Front Surg. 2026 May 28;13:1820935. doi: 10.3389/fsurg.2026.1820935 (PMC13254199; doi:10.3389/fsurg.2026.1820935)
Supplement: Supplementary file 1 [file Supplementaryfile1.docx]

**Supplementary Table 1. Clinical characteristics and outcomes of patients with De Novo Pulmonary vein stenosis**

| Patient | Lesion | Diagnosis Timing | D-R Weight Ratio | Clinical Category | Course | Intervention | Outcome |
| --- | --- | --- | --- | --- | --- | --- | --- |
| 1 | Bilateral | 3 months | 1.94 | Significant | Progressive | Yes | Mortality |
| 2 | Bilateral | 2 months | 2.81 | Significant | Progressive | Yes | Survival |
| 3 | Right | 36 months | 1.96 | Subclinical | Stable | No | Survival |
| 4 | Right | 0 month | 2.7 | Subclinical | Stable | No | Mortality |
| 5 | Right | 4 months | 1.43 | Subclinical | Stable | No | Survival |
| 6 | Right | 14 months | 1.48 | Subclinical | Stable | No | Survival |
| 7 | Right | 0 month | 1.68 | Subclinical | Improved | No | Mortality |
| 8 | Right | 3 months | 1.69 | Subclinical | Stable | No | Survival |
| 9 | Left | 3 months | 2.54 | Subclinical | Stable | No | Survival |

*D-R: Donor-Recipient*
